# CBS-Induced H_2_S Generation in Hippocampus Inhibits EA-Induced Analgesia

**DOI:** 10.1155/2020/5917910

**Published:** 2020-04-29

**Authors:** Wen-Jing Ren, Jia Fu, Hai-Yan Yin, Neng-Gui Xu, Chun-Zhi Tang, Li-Zhou Liu, Shu-Guang Yu, Yong Tang

**Affiliations:** ^1^School of Acupuncture and Tuina, Chengdu University of Traditional Chinese Medicine, Chengdu, Sichuan, China; ^2^Acupuncture & Chronobiology Key Laboratory of Sichuan Province, Chengdu, Sichuan, China; ^3^College of Medicine, Chengdu University, Chengdu, Sichuan, China; ^4^School of Acupuncture and Tuina, Guangzhou University of Chinese Medicine, Guangzhou, China; ^5^Centre for Health, Activity, and Rehabilitation Research, School of Physiotherapy, University of Otago, Dunedin, New Zealand

## Abstract

Hydrogen sulfide (H_2_S) is an important mediator participating in both physiological and pathological systems and related to the inflammatory process. Acupuncture has a therapeutic effect on inflammatory pain. However, whether H_2_S generated in the central nervous system (CNS) is a mediator of electroacupuncture (EA) treatment for inflammatory pain is unknown. We injected complete Freund's adjuvant (CFA) to induce inflammatory pain and applied EA treatment as an interventional strategy for pain relief. The results presented here show that S-adenosyl-l-methionine (SAM), an allosteric activator of cystathionine-*β*-synthetase (CBS), may reverse the therapeutic effect of EA. CBS-induced H_2_S generation might get involved in the mechanism of EA-induced analgesia in the hippocampus on chronic inflammatory pain.

## 1. Introduction

Hydrogen sulfide (H_2_S) is the most recently accepted endogenously produced gasotransmitter that plays an important role in human health and physiology. Emerging evidence indicates that physiological concentrations of H_2_S might have an anti-inflammatory effect, while higher concentrations can exert proinflammatory effects [1]. H_2_S is synthesized by three enzymes: cystathionine *γ*-lyase (CSE), cystathionine *β*-synthetase (CBS), and 3-mercaptopyruvate sulfurtransferase (3-MST) [2]. The expression of CBS, CSE, and 3-MST shows tissue-specific dominance. Both CBS and 3-MST, as the primary physiological source of H_2_S in the central nervous system, predominantly are localized in the brain; CSE abundantly exists at the vascular or nonvascular smooth muscle in the mammalian cardiovascular and respiratory system; therefore, H_2_S-producing enzymes are generally ubiquitously expressed in mammalian tissues and impact a wide range of cellular processes [3, 4]. Homocysteine and cysteine are substrates for H_2_S production, and H_2_S-synthesizing enzymes are responsible for converting these substrates to H_2_S; in the presence of cysteine, especially homocysteine, CBS catalyzes the production of H_2_S [5].

H_2_S and its synthesizing enzymes got involved in a variety of conditions, including pain and inflammation [6]. It has been indicated that H_2_S has an effect on pro- and anti-inflammation, and it is a mediator of peripheral and neurogenic inflammation [1]. The same as inflammation, H_2_S participating in modulating visceral pain is also controversial [6]. H_2_S-generating enzyme CBS is also involved in several pain conditions. CBS has been found with an increased expression in the dorsal root ganglion (DRGs) of the IBS model induced by neonatal colonic inflammation (NCI) [7]. Inhibition of the CBS-H_2_S signaling pathway markedly attenuated heterotypical intermittent stress-induced visceral hyperalgesia; particularly, aminooxyacetic acid (AOAA), one of the CBS inhibitors, suppressed voltage-gated sodium channel currents of the colon-specific DRG neurons and reversed the enhanced expression of NaV1.7 and NaV1.8 subtypes [8]. These findings indicated that CBS plays a crucial role in visceral hyperalgesia. Furthermore, CBS expression can be also upregulated in neuropathic pain and inflammatory pain. Chronic construction injury (CCI) surgery caused significantly increased expression of CBS in the ipsilateral ventral and dorsal horn of L4-6 spinal cord segments [9]. Inhibiting CBS by intrathecal injection of AOAA attenuated mechanical and thermal hypersensitivity, and AOAA injecting suppressed the expression of Na_V1.7_ and Na_V1.8_ in DRG neurons [10]. Additionally, application of AOAA was observed to alleviate CFA-induced hyperalgesia on the temporomandibular joint and reduce CBS-induced H2S production in trigeminal ganglion by reversing the enhancement of neural hyperexcitability and increasing the voltage-gated potassium currents [11]. These findings suggest that H_2_S and CBS could play a pivotal role in a variety of pain conditions.

Acupuncture has been applied in various pain conditions, and the neurobiological mechanism has been the important focus to be investigated [12, 13]. When the needle is connected with an electrode, it is called as electroacupuncture (EA). EA is also very popular in the basic research and clinic practice because the stimulus is under control [14, 15]. EA stimulation has been found to induce time-dependent cumulative analgesia in rats with neuropathic pain, and the mechanism was demonstrated to be related to the activation of hippocampal MEK1 [16]. Recent studies have also shown that repeated EA intervention leads to synaptic remodeling of hippocampal neurons, and in consequence, the expression of calcium/calmodulin kinase II in the hippocampal CA3 region is upregulated, which contributed to acupuncture analgesia [17]. However, whether H2S induced by CBS in the hippocampus would be able to get involved in acupuncture analgesia still remains unclear. In this study, we hypothesized that EA might modulate the H2S generation induced by CBS in the hippocampus to alleviate chronic inflammatory pain.

## 2. Materials and Methods

### 2.1. Animals

Male BALB/c mice (8–9 weeks, 20–23 g) were purchased from Chengdu Da Shuo Experimental Animals Co. Ltd. All mice were adapted to the standard laboratory conditions (24 ± 2°C room temperature and 65 ± 5% humidity on 12/12 h light-dark cycles) with drinking water and food available *ad libitum*. The experimental procedures were conducted in accordance with the National Institutes of Health (NIH) Guidelines for the Care and Use of Laboratory Animals and approved by the Animal Ethics Committee of Chengdu University of Traditional Chinese Medicine. After adaptive domestication for one week, mice were randomly divided into different groups based on random numbers generated by SPSS software.

### 2.2. Chronic Inflammatory Pain Model

Mice were anesthetized with 1% isoflurane, and complete Freund's adjuvant (CFA, 20 *μ*l) or saline (20 *μ*l) was subcutaneously injected into the right hind paw [18]. Thermal hyperalgesia was detected at the first day after injection [19].

### 2.3. EA Treatment

An electroacupuncture apparatus (HANS-200A acupoint nerve stimulator, Nanjing Jisheng Medical Co., Ltd) was used for EA treatment. EA stimulation was provided at the first day after CFA injection and subsequently lasted for one week. Mice were shaved to expose ST36, located at the posterolateral part of the knee about 2 mm below the fibular head [20], and two stainless steel needles (0.18 mm in diameter, 13 mm in length) were inserted directly at ST36 of both hind limbs about 3 mm deep. The stimulated parameters of EA included 0 Hz, 2 Hz, 100 Hz, and 2/100 Hz (0.2 mA intensity), lasting for 30 min during one session. During treatment, all mice were restrained on a specific device [21].

### 2.4. Intraperitoneal Injection

Mice were randomly divided into seven groups: CFA + Saline (*n* = 6), CFA + NaHS (*n* = 6), CFA + HA (*n* = 7), CFA + AOAA (*n* = 6), CFA + SAM (*n* = 7), CFA + IC2A (*n* = 7), and CFA + L-aspartate (*n* = 6). Saline, NaHS (H_2_S donor, 5.6 mg/kg), hydroxylamine (HA, one of CBS inhibitors, 50 mg/kg), AOAA (one of CBS inhibitors, 45 mg/kg), SAM (CBS agonist, 100 mg/kg), and IC2A (7.9 mg/kg) or L-aspartate (1.25 mg/kg) were injected intraperitoneally one day after CFA injection and lasted for one week. No abnormalities were observed during the period of injection.

### 2.5. Surgical Procedures and Microinjection

Stereotaxic surgery was performed 7 days prior to experiments. Mice were anesthetized with isoflurane and fixed on a stereotaxic platform. Coordinates of A-P: 2.5 mm relative to the bregma, lateral: ±2.5 mm relative to the midline, and depth: 2 mm from the duramater were used for implanting guide cannulas (RWD, China) into the bilateral hippocampi [22]. After recovery from surgery, HA (9 nmol/1 *μ*l [23]), AOAA (9 nmol/1 *μ*l [24, 25]), or SAM (10 pmol/1 *μ*l [26]) was bilaterally microinjected into the hippocampi. DAO and CAT inhibitors followed the same procedure. A 5 *μ*l microsyringe was held by a micromanipulater on the stereotaxic apparatus (RWD, China), and injection was performed at a rate of 0.5 *μ*l/min. The volume of each microinjection was 1 *μ*l. Afterwards, the needle was kept at the injection place for another 5 minutes after each injection to allow for sufficient diffusion. No adverse effect was observed during each injection.

### 2.6. Thermal Withdrawal Latency (TWL)

Mice were placed in behavior boxes on a glass platform before the behavior test [27]. Thermal hyperalgesia was assessed by measuring the TWL with a Plantar Test Apparatus (10% light intensity, 10 sec baseline latency, and 20 sec cutoff time. Hargreaves method, PL-200, Tai Meng, China). After 30-min of acclimatization, a mobile radiant heat source was focused on the plantar surface of the right hind paw. Each mouse was tested three times with intervals of 5 min. Pain threshold was tested as the baseline at day 1 before CFA injection and day 1, 3, 5, and 7 after CFA injection. In order to eliminate observational bias, all behavioral data were collected and recorded by the same investigator who was not responsible for drug injection or EA treatment.

### 2.7. Drugs

Complete Freund's adjuvant (CFA, F5881), sodium hydrogen sulfide (NaHS, 161527), hydroxylamine (HA, 438227), aminooxyacetic acid (AOAA, C13408), and L-aspartate (A9256) were purchased from Sigma (Sigma-Aldrich St. Louis, MO, USA). IC2A (I129126) was purchased from Aladin (Shanghai Aladin Biochemical Technology Co. LTD). S-adenosine-L- methionine (SAM, S9990) was purchased from Solarbio (Beijing Solarbio Technology Co. LTD). Saline was purchased from Kelun (Sichuan Kelun Pharmaceutical Co. Ltd.).

### 2.8. Statistical Analysis

All data were analyzed and graphed using Graphpad Prism 6 (GraphPad Software, Inc., La Jolla, CA, USA). Data were expressed as data = MEANS ± SEM (standard error of means). Two-way repeated-measure ANOVA followed by the Tukey–Kramer test was performed to compare the differences between groups. A value of *P* < 0.05 was considered statistically significant.

## 3. Results

### 3.1. Low-Frequency EA Stimulation Alleviated Chronic Inflammatory Pain

**Figure 1:**
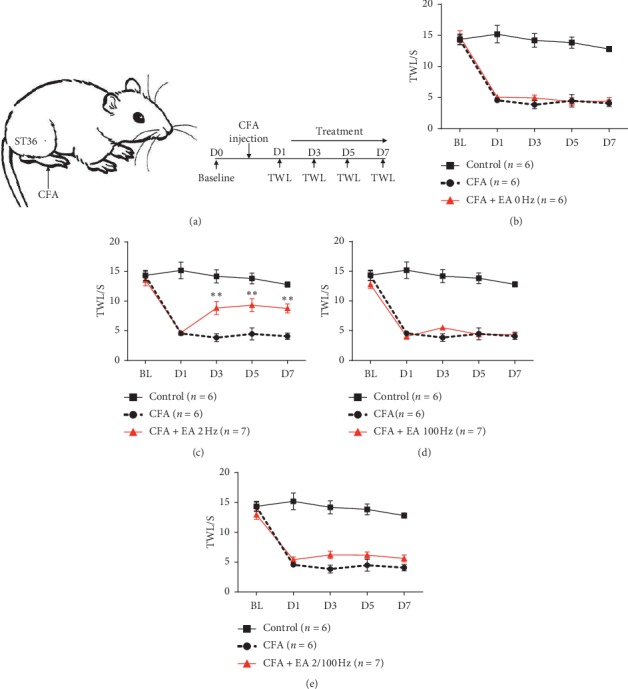
EA at a low frequency of 2 Hz had analgesic effects on thermal hyperalgesia in mice with inflammatory pain. (a) Schematic drawing for CFA injection and the location of acupoint ST36 in a mouse. (b) EA (0 Hz) had no significant therapeutic effect on chronic inflammatory pain (P > 0.05); F(4, 40) 0.3040, *P* = 0.8736 (interaction), F(4, 40) 123.7, P < 0.0001 (time), and F(1, 10) 0.6222, *P* = 0.4485 (column factor). (c) Chronic inflammatory pain could be alleviated by EA at low frequency (2 Hz) (P < 0.01); F(4, 44) 8.287, P < 0.0001 (interaction), F(4, 44) 53.98, P < 0.0001 (time), and F(1, 11) 13.39, *P* = 0.0038 (column factor). (d) EA at 100 Hz failed to relieve hyperalgesia (P > 0.05); F (4, 44) 2.366, P 0.0674 (interaction), F (4, 44) 116.6, P < 0.0001 (time), and F (1, 11) 0.008546, *P* = 0.9280 (column factor). (e) EA at 2/100 Hz failed to relieve hyperalgesia (P > 0.05); F (4, 44) 3.416, *P* = 0.0162 (interaction), F (4, 44) 98.86, P < 0.0001 (time), and F (1, 11) 2.898, *P* = 0.1167 (column factor). Data MEANS± SEM

To investigate the efficacy of EA in chronic inflammatory pain, we injected CFA in the right plantar surface of the hind paw and measured responses to the thermal stimuli in separate groups at a different time point. We chose the ST36 acupuncture point, which is one of the most frequently used acupoints for pain relief [28, 29] (Figure 1(a)). After one day, different groups received acupuncture treatments with different stimulated parameters at ST36, and then we measured changes of TWL on mice (Figures 1(b)–1(e)). On the first day after CFA injection, we found that the baseline value of TWL was significantly decreased (Figures 1(b)–1(e)). On the 3rd day after acupuncture treatment, EA at low frequency (2 Hz) had an analgesic effect on thermal hyperalgesia compared with EA at high-frequency stimulation(100 Hz) and alternating frequency(2/100 Hz) on mice with inflammatory pain (*P* < 0.05) (Figures 1(c)–1(e)). In addition, acupuncture without electrostimulation also failed to alleviate pain sensation (*P* > 0.05) (Figure 1(b)). The result suggests that low-frequency EA has a therapeutic effect on CFA-induced inflammatory pain, but acupuncture and high or alternating frequency EA stimulation failed to alleviate pain.

### 3.2. Intraperitoneal Injection of CBS Inhibitors Attenuated Thermal Hypersensitivity

**Figure 2:**
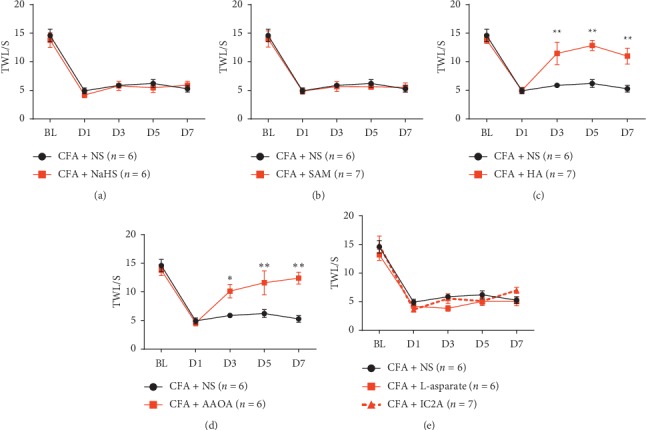
Inhibiting CBS activity alleviated pain sensation on the CFA mouse model. (a) H2S donor NaHS failed to reduce thermal pain behavior(P > 0.05); F (4, 40) 0.3923, *P* = 0.8129 (interaction), F (4, 40) 62.42, P < 0.0001 (time), and F (1, 10) 0.2892, *P* = 0.6025 (column factor). (b) CBS agonist SAM failed to attenuate thermal pain behavior(P > 0.05); F (4, 44) 0.08392, P 0.9870 (interaction), F (4, 44) 42.73, P < 0.0001 (time), and F (1, 11) 0.3571, *P* = 0.5622 (column factor). (c) HA notably attenuated thermal hypersensitivity (P < 0.01); F(4, 44) 6.669, *P* = 0.0003 (interaction), F(4, 44) 24.06, P < 0.0001 (time), and F (1, 11) 18.68, *P* = 0.0012 (column factor). (d) AOAA also remarkably relieved pain behavior (P < 0.05); F (4, 40) 7.503, *P* = 0.0001 (interaction), F (4, 40) 27.84, P < 0.0001 (time), and F (1, 10) 12.95, *P* = 0.0049 (column factor). (e)Theinjection of IC2A and L-aspartate had no effect on relieving thermal pain(P > 0.05); F(8, 64) 0.8894, *P* = 0.5306 (interaction), F(4, 64) 81.15, P < 0.0001 (time), and F (2, 16) 2.716, *P* = 0.0965 (column factor). Data MEANS± SEM

To study the relationship between H_2_S and inflammation, NaHS (5.6 mg/kg [30, 31]) was intraperitoneally injected one day after CFA injection, and results indicated that NaHS failed to alleviate pain behavior (Figure 2(a)). Similar to NaHS, SAM (100 mg/kg) [32, 33] had no therapeutic effect on inflammatory pain (Figure 2(b)). However, HA (50 mg/kg) [34] and AOAA (45 mg/kg) [34] attenuated thermal hypersensitivity (Figures 2(c)–2(d)). A recent finding indicated that H_2_S could also be produced by 3-MST from 3-mercaptopyruvate in the brain [35, 36]. Therefore, we applied a specific cysteine aminotransferase (CAT) inhibitor L-aspartate and D-amino acid oxidase (DAO) inhibitor IC2A [37]. However, they also failed to alleviate inflammatory pain (Figure 2(e)). These results indicated that CBS-induced H_2_S induced the pain behavior and CBS inhibitors could alleviate pain sensation.

### 3.3. Hippocampal Injection of CBS Inhibitors Attenuated Thermal Hypersensitivity

**Figure 3:**
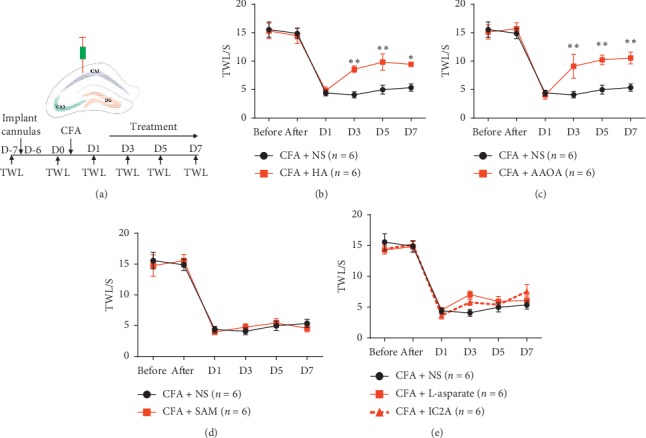
Inhibiting CBS activity in the hippocampus alleviated pain sensation on the CFA mice model. (a) Schematic drawing of microinjection in the hippocampus. (b) The injection of HA notably attenuated thermal hypersensitivity (P < 0.05); F (5, 50) 3.790, *P* = 0.0055 (interaction), F (5, 50) 50.14, P < 0.0001 (time), and F (1, 10) 10.78, *P* = 0.0082 (column factor). (c) AOAA remarkably relieved pain behavior (P < 0.05); F (5, 50) 3.760, *P* = 0.0057 (interaction), F (5, 50) 40.77, P < 0.0001 (time), and F (1, 10) 13.44, P 0.0043 (column factor). (d) SAM failed to alleviate thermal pain behavior (P > 0.05); F (5, 50) 0.3387, *P* = 0.8870 (interaction), F (5, 50) 78.47, P < 0.0001 (time), and F (1, 10) 0.004093, *P* = 0.9502 (column factor). (e) IC2A and L-aspartate injection failed to alleviate thermal pain behavior (P > 0.05); F (10, 75) 1.313, *P* = 0.2394 (interaction), F (5, 75) 125.1, P < 0.0001 (time), and F (2, 15) 1.092, *P* = 0.3608 (column factor). Data MEANS± SEM

To study the relationship between CBS-induced H_2_S in the brain and thermal hypersensitivity, the CBS inhibitor HA (9 nmol) [23] or AOAA (9 nmol) [24, 25] was bilaterally microinjected into the hippocampus. Moreover, the CBS-specific agonist SAM (10 pmol) [26] or saline was also bilaterally injected into the hippocampus one day after CFA injection, and CAT and DAO inhibitors [37] followed the same procedure (Figure 3(a)). Results showed that HA and AOAA microinjection significantly increased the TWL and attenuated thermal hypersensitivity (Figures 3(b) and 1(c)). However, saline, SAM, IC2A, or L-aspartate injection failed to alleviate thermal pain behavior (Figures 3(d) and 3(e)). Therefore, suppressing H_2_S induced by CBS in the hippocampus might relieve CFA-induced chronic inflammatory pain.

### 3.4. SAM Reversed the Therapeutic Effect of Low-Frequency EA

**Figure 4:**
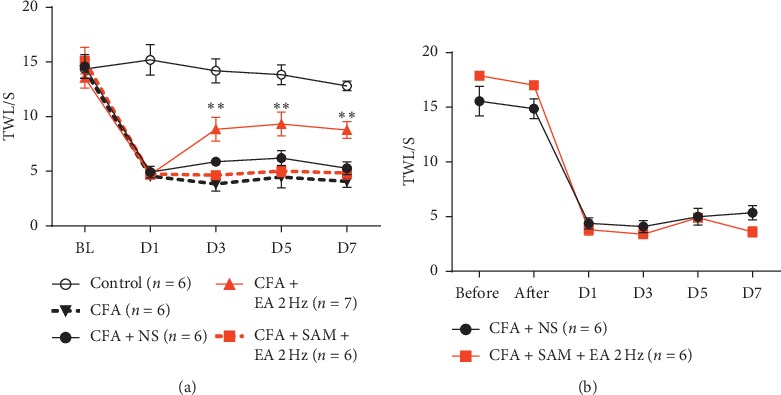
Treatment of CBS agonist SAM reverses the therapeutic effect of EA. (a) Compared with EA(2 Hz), EA failed to alleviate pain sensation after SAM intraperitoneal injection (*P* < 0.01); *F* (8, 64) = 3.965, *P*=0.0007 (interaction), *F* (4, 64) = 77.51, *P* < 0.0001 (time), and *F* (2, 16) = 7.916, *P*=0.0041 (column factor). (b) EA also failed to alleviate pain sensation after SAM microinjection (*P* > 0.05); *F* (5, 50) = 3.571, *P*=0.0077 (interaction), *F* (5, 50) = 202.1, *P* < 0.0001 (time), and *F* (1, 10) = 0.2729, *P*=0.6128 (column factor). Data = MEANS ± SEM.

To determine whether CBS influences EA therapeutic effect on chronic inflammatory pain, EA intervention(2 Hz, 0.2 mA) was performed immediately after SAM injection or microinjection. However, EA stimulation failed to alleviate thermal pain behavior after SAM injection (Figures 4(a) and 4(b)). These results indicated that activating CBS reversed EA therapeutic effect on inflammatory pain, and CBS-induced H_2_S release might inhibit EA therapeutic effect.

## 4. Discussion

Our data demonstrated that low-frequency EA stimulation notably alleviated the thermal pain behavior, and injection of CBS inhibitors might have a therapeutic effect on thermal pain. As an essential synthetase of H_2_S, CBS is mostly responsible for the production of H_2_S in brain tissue and is highly expressed in the hippocampus [38, 39]. Therefore, H_2_S generation in the hippocampus might participate in the CFA-induced chronic inflammatory pain. Our data further showed that SAM, a CBS agonist, could reverse EA therapeutic effect on chronic inflammatory pain. Taken together, the downregulation of CBS-induced H_2_S generation might be one of the mechanisms of EA treatment for chronic inflammatory pain.

H_2_S is the third gasotransmitter discovered after NO, CO. It is considered as an important signaling molecule in the regulation of pathophysiological processes, including pain and inflammation [34, 40, 41]. However, the precise role of H_2_S in inflammation is not clear. It was supposed to have pro- or anti-inflammatory effects under different conditions. In general, H_2_S at physiological concentrations might have an anti-inflammatory effect, while increased concentrations of H_2_S can exert proinflammatory effects [1]. Intraplantar administration of NaHS, the donor of H_2_S, evoked mechanical hyperalgesia [42]. It was found that H_2_S might activate Cav3.2 T-type Ca2+ channels that led to sensitization of nociceptive processing and hyperalgesia [43]. H_2_S synthetase CSE and CBS also play a pivotal role in pain and inflammation. Pretreatment with the CSE inhibitor propargylglycine significantly reduced hind paw edema and decreased granulocyte infiltration into the tissue in response to an injection of carrageenan [44]. In the visceral pain rat model, the expression of CBS in the spinal cord was notably increased [45]. Some investigations also indicated that CBS was upregulated by TLR4 in NCI rats and mediated by the NF-*κ*B signaling pathway, thus contributing to visceral hypersensitivity [46]. In addition, the expression of CBS also has an effect on inflammatory pain. It was found that the expression of CBS in the rat dorsal root ganglia (DRG) was significantly upregulated after intraplantar administration of CFA, and the mechanical hyperalgesia was expectedly attenuated by intraperitoneal injection of CBS inhibitors in a dose-dependent manner [34]. These investigations suggest that H_2_S and its enzymes in the peripheral nervous system are regulated by nociception. However, analgesic effect of H_2_S and its enzymes on the brain are yet to be investigated in future studies.

CBS is responsible for most of the H_2_S production in the brain, and it is highly expressed in the hippocampus. Multiple evidences indicated that the hippocampal formation responded to external nociceptive stimuli [47–49]. Previously, investigations showed that SAM resulted in an up to eight or ten-fold increase of the enzymatic activity of CBS [50–52], and CBS inhibitors HA and AOAA suppressed H_2_S production in brain homogenates which can be enhanced by the CBS activator [39]. In addition, several investigations found that intraperitoneal injection of CBS inhibitors could decrease CBS expression and activity in brain tissue. For instance, Liu and his colleagues found that intraperitoneal injection of HA inhibited CBS protein expression as examined by western blot analysis and immunohistochemical analysis [53]. AOAA (i.p) also reduced CBS activity in brain tissue [54]. Thus, in order to study the relationship between CBS-induced H_2_S and inflammatory pain, HA or AOAA was intraperitoneally injected into mice. Results has shown that injecting HA or AOAA led to relieve pain by inhibiting CBS. It is in consistent with the previous research [34]. However, analgesic effect of CBS-induced H_2_S on the brain is yet to be investigated in future studies. To further investigate whether CBS located in the hippocampus would affect pain sensation, HA or AOAA was bilaterally microinjected into the hippocampus. We found that inhibiting H_2_S generation in the hippocampus alleviated inflammatory pain and activated H_2_S generation by microinjected SAM had no effect on inflammatory pain. However, how CBS in the hippocampus participates in pain sensation is yet to be investigated. According to previous studies, CBS converted homocysteine to cysteine [55]. CBS inhibitors could promote accumulation of L-homocysteine and L-cysteine. It was reported that reducing L-cysteine and L-homocysteine induced time and dose-dependent peripheral hyperalgesia which could be modulated by 3bOH, a potent T-type Ca2+ channel blocker [56]. Therefore, homocysteine and cysteine might participate in inflammatory pain process, but it also needs to be further investigated.

Acupuncture has a therapeutic effect on inflammatory pain, and EA therapy has a cumulative effect [16, 57, 58]. The frequency of EA is one of the crucial factors to significantly influence its therapeutic effects. EA with different frequencies is regarded as distinct therapeutic methods and have been used in different diseases. High-frequency (100 Hz) stimulation was more effective as compared with the low frequency (2 Hz) in amelioration of muscle spasticity, and it might be mediated by dynorphin generating from the central nervous system [59]. Low-frequency EA released *β*-endorphin and enkephalins, while high-frequency EA released the dynorphins to suppress pain sensation [60]. However, low-frequency EA produced the significant rapid decrease in ankle edema after ankle sprain which is an important feature of inflammation [61]. Furthermore, low-frequency EA (10 Hz) significantly reduced CFA-induced hind paw edema to suppress inflammation by activating the hypothalamus-pituitary-adrenal axis (HPA); however, 100 Hz EA had no effect on it [62]. Some investigations demonstrated that low-frequency EA could regulate the expression level of genes in the nucleus arcuate region, and low-frequency EA at ST36 in rats markedly relieved pain [63, 64]. Low-frequency EA could also suppress spinal long-term potential (LTP) to alleviate pain sensation in colitis rats [65]. Additionally, low-frequency stimulation, rather than high frequency, invoked endogenous mechanisms for the release of 5-hydroxytryptamine (5-HT) in the spinal cord, which activated 5-HT receptors to reduce hyperalgesia in rats with joint inflammation [66]. In accordance with these results, we found that low-frequency EA delivered to ST36 could alleviate pain. It has been reported that EA applied to ST36 could influence hemodynamic signaling in the hippocampus [42]. Additionally, the previous report indicated that EA protected against hypoxic ischemic brain damage in immature rats via decreasing H_2_S generation in brain tissue [53]. Also, EA treatment decreased CBS expression level and increased HO-1 and HIF-1*α* expression levels in perinatal rat cortex cells [67]. Thus, EA protected against hypoxic damage via the hydrogen sulfide/CBS-CO/HO-1-HIF-1*α* system.

To further determine the role of H_2_S in EA stimulation, EA treatment was performed after injection of the CBS agonist SAM on a CFA-induced inflammatory mouse model, and we found that the EA therapeutic effect was suppressed by SAM. H_2_S was expected to play an important role in modulating EA effect on inflammatory pain. Although the H_2_S/CBS system participates in the inflammatory pain and it is related to EA treatment, the mechanisms of how EA modulates H_2_S release need to be confirmed in our future work. Previous research has shown that intracellular Ca^2+^ can be regulated by the cAMP/PKA pathway, and H_2_S can regulate [Ca^2+^]_i_ through the cAMP/PKA system [68]. EA pretreatment could produce an antiarrhythmic effect by regulating the L-type Ca^2+^ channel [69]. Additionally, AOAA, an inhibitor of CBS, could also suppress the potentiation of ATP-induced intracellular calcium signals in DRG neurons to markedly attenuate pain hypersensitivity [70]. Some investigators also showed that EA pretreatment could inhibit Ca^2+^ influx to alleviate the LPS-induced inflammation in rats [71]. Therefore, we speculated that acupuncture regulation of H_2_S production might be related to calcium channel opening. This needs to be proved in our future work.

## 5. Conclusions

Current data indicated that the CBS-specific agonist SAM may reverse the therapeutic effect of EA. Downregulation of CBS might be a mediator of EA-induced analgesia on chronic inflammatory pain.

## Data Availability

The data used to support the findings of this study are available from the corresponding author upon request.
